# Intravitreal Dexamethasone in Patients with Wet Age-Related Macular Degeneration Resistant to Anti-VEGF: A Prospective Pilot Study

**DOI:** 10.1155/2018/5612342

**Published:** 2018-07-29

**Authors:** Ermete Giancipoli, Antonio Pinna, Francesco Boscia, Gianluigi Zasa, Giovanni Sotgiu, Simone Dore, Giuseppe D'Amico Ricci

**Affiliations:** ^1^Department of Biomedical Sciences, University of Sassari, Sassari, Italy; ^2^Department of Medical, Surgical, and Experimental Sciences, Ophthalmology Unit, University of Sassari, Sassari, Italy; ^3^Azienda Ospedaliero-Universitaria (AOU) di Sassari, Sassari, Italy; ^4^Department of Biomedical Sciences, Clinical Epidemiology and Medical Statistics Unit, University of Sassari, Sassari, Italy

## Abstract

**Purpose:**

To evaluate the efficacy and safety of a single intravitreal dexamethasone implant (DXI) combined with intravitreal antivascular endothelial growth factor (anti-VEGF) therapy, in patients with neovascular age-related macular degeneration (wet-AMD) resistant to conventional treatment.

**Methods:**

In this randomized, controlled pilot study, 16 eyes of 15 patients, unresponsive to anti-VEGF therapy, were enrolled and randomly assigned to two groups: DXI + anti-VEGF (treatment group: 11 eyes) and monthly anti-VEGF alone (control group: 5 eyes). Patients were treated at baseline and followed for 6 months. Best corrected visual acuity (BCVA), optical coherence tomography (OCT) parameters, and fluorescein angiography (FA) were evaluated.

**Results:**

Eight eyes (72.7%) in the treatment group and 2 eyes in the control group (40%) showed complete retinal fluid resorption (*p*=0.049). BCVA showed no significant change from baseline in both the treatment group and the control group (*p*=0.40 and *p*=0.29, respectively). Both median central foveal thickness (CFT) and median macular volume showed a greater reduction from baseline in the treatment group.

**Conclusion:**

In patients showing an incomplete response to anti-VEGF therapy, DXI combined with intravitreal anti-VEGF seems to improve retinal fluid resorption without functional advantage. This trial is registered with ACTRN12618001102268.

## 1. Introduction

The advent of antivascular endothelial growth factor (anti-VEGF) intravitreal therapy introduced a new standard of care for patients with neovascular age-related macular degeneration (wet-AMD). Although anti-VEGFs are effective to prevent severe visual loss in most cases, often promoting a significant visual improvement, there are some patients with wet-AMD who continue to experience a visual deterioration despite an adequate treatment [1]. Results from clinical trials have revealed that more than 50% of patients, treated with monthly intravitreal ranibizumab or bevacizumab for two years [2], and up to 33% of patients, treated with aflibercept 2.0 mg [3], showed persistent retinal fluid on optical coherence tomography (OCT). Long-lasting intraretinal or subretinal fluid (IRF/SRF) may induce irreversible damage to retinal structures, preventing optimal visual recovery [4]. Moreover, the need for frequent treatments for prolonged period adds substantial burdens and safety concerns for these patients [5].

Inflammation is involved in both the beginning and the progression of AMD [6, 7]. To counteract, inflammation could lead to a better control of this pathology. The complementary action of intravitreal steroid injections in wet-AMD dates back to the combination of intravitreal triamcinolone acetonide (TA) with photodynamic therapy (PDT) [8–10]. In the last decade, a sustained-release 700 *µ*g dexamethasone intravitreal implant (DXI; Ozurdex; Allergan, Irvine, California, USA) has been approved for the treatment of macular oedema secondary to retinal vein occlusions (RVOs), noninfectious uveitis, and diabetic macular oedema [11, 12].

The purpose of this study was to evaluate the anatomical and visual outcomes in patients with wet-AMD and persistent IRF/SRF, after adding DXI to the already ongoing anti-VEGF therapy. To the best of our knowledge, this is the first controlled, pilot study assessing DXI as an adjunctive therapy for patients with refractory wet-AMD.

## 2. Methods

This controlled, pilot study was conducted at the Ophthalmology Unit, University of Sassari, Italy, between March and October 2016. The study protocol adhered to the tenets of the Declaration of Helsinki, and Ethics Committee approval was obtained. All patients provided written informed consent.

We enrolled only patients diagnosed with subfoveal AMD-related CNV with evidence of persistent IRF/SRF, despite at least 4 consecutive monthly injections of anti-VEGF agents, administered just before inclusion in the study. Exclusion criteria are listed in Table 1.

Patients were randomized into two groups: DXI + anti-VEGF and monthly anti-VEGF alone. The arm that received DXI was defined as the treatment group, while the arm receiving anti-VEGF monotherapy was labeled as the control group. All patients underwent a baseline examination, during which the assigned treatment (computer-generated randomization) was administered, and were then evaluated at days 15, 30, 60, 90, 120, and 180. At each follow-up, slit lamp examination, distance best corrected visual acuity (BCVA), intraocular pressure (IOP) measurement by Goldmann applanation tonometry, dilated fundus examination, and SD-OCT were performed. Snellen BCVA was converted to LogMAR for statistical purposes.

OCT examination was carried out with Heidelberg Spectralis HRA (Heidelberg Engineering, Heidelberg, Germany); radial scan (24 sections at 6/frame rate of 30° length and 7.5° apart) and volume scan (20° × 20° at high resolution with a 5/frame rate and 49 sections) were obtained for each patient. Every single image was revised for quality and graded by two different physicians (GDR and EG). Each B-scan composing volume pattern was evaluated and manually resegmented, considering the outer aspect of retinal pigment epithelium (RPE) as the outer border and the internal limiting membrane (ILM) as the inner border, in order to obtain a standardized layer segmentation. Each radial B-scan was then assessed for integrity of the outer retina. Any discontinuity of the hyperreflective line referred as inner segment/outer segment (IS/OS) junction, external limiting membrane (ELM), and RPE was identified and measured. The values from each B-scan were than averaged in order to estimate the global extension of the outer retina damage for each eye. These estimates were carried out on month 2 OCT, when the IRF/SRF reduction was greater and the visualization of external layers improved.

Fluorescein angiography (FA) was performed at baseline and at 2 months with Heidelberg Spectralis HRA. Fundus autofluorescence (FAF) images were obtained at baseline and at the end of the follow-up with Heidelberg Spectralis HRA, as well.

Patients belonging to the treatment group were treated with a single DXI injection at baseline, as an adjunctive treatment to the ongoing anti-VEGF therapy (ranibizumab 0.5 mg or aflibercept 2 mg). Starting from month one, the treatment group continued anti-VEGF therapy according to an as-needed regimen (retreatment criteria in Table 1), whereas patients in the control group were evaluated and treated on a monthly basis, for 6 months. No further injections of DXI were administered during the study.

The primary outcome was the complete regression of IRF/SRF at SD-OCT (no evidence of any fluid at each B-scan in both radial and volume patterns). Secondary outcomes were as follows: the safety profile of the treatment, the change of median BCVA, median CFT, median macular volume at OCT, and leakage area at the FA examination during the follow-up.

We also investigated if the extension of IS/OS, RPE, and ELM damage at OCT could correlate with median BCVA at month 2 and with the pattern of fluid.

### 2.1. Statistical Analysis

Descriptive statistics of the categorical variables were performed using absolute frequency and percentage, while continuous variables were summarized as mean (standard deviation—SD) or median (interquartile range—IQR), when appropriate. The inferential analysis of quantitative variables was performed with the chi-squared test or Fisher's exact test. After the assessment of their distribution with the Shapiro–Wilk normality test, the differences between continuous variables were computed with the student's *t*-test or the Wilcoxon test, when appropriate. CFT, BCVA, and IOP at each time point of follow-up were compared with their respective baseline values using the Wilcoxon matched pairs test.

The survival analysis with the Kaplan–Meier curves followed by the log-rank test was performed to evaluate the eyes that developed a complete response and those that did not in the treatment and control groups, respectively. A *p* value < 0.05 was considered to be statistically significant. The statistical analysis was carried out using Stata software (Stata/MP 13.0 for Mac, StataCorp, College Station, TX). All data collected will be available on request.

## 3. Results

Sixteen eyes (8 right eyes, 8 left eyes) of 15 consecutive patients (5 females, 10 males) with a mean (SD) age of 75 (8.8) years were enrolled. For the only patient whose eyes were both eligible, we randomly assigned one eye to the treatment group and the other to the control group. Seven eyes were pseudophakic (3 in the control group and 4 in the study group), and no patient underwent any kind of surgery during the study period. Patients received a mean (SD) number of 8.9 (3.8) injections before entering the study. Fifteen eyes had been treated with ranibizumab and 1 eye with aflibercept before enrollment, and they were kept on the same drug during the study. Four patients (25%) were defined as initial nonresponders, as they showed no BCVA improvement and no CFT decrease after four initial monthly injections. Eleven patients (12 eyes, 75%) were classified as late nonresponders since they showed a complete response during the very first period of their treatment, without exhibiting the same response in the last period.

Eye distribution in the two groups was as follows: 11 in the treatment group and 5 eyes in the control group. There were no significant differences between the groups at baseline (Table 2). There was no dropout during the follow-up period.

**Figure 1:**
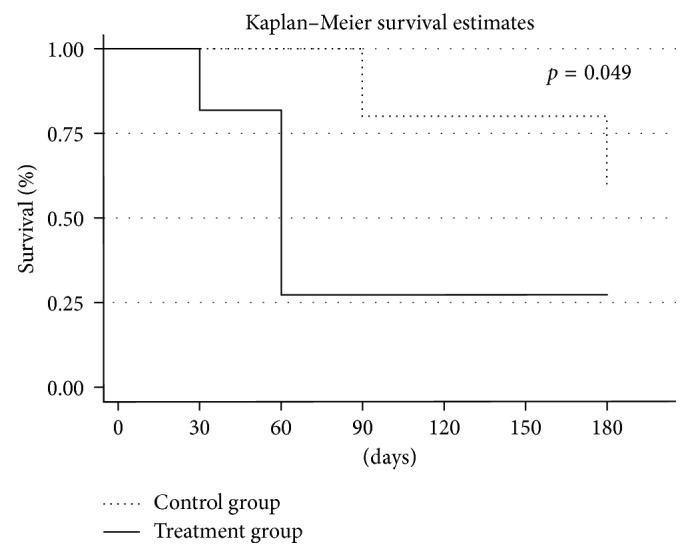
Kaplan–Meier survival curve: each line shows the proportion of patients in each group (solid line: treatment group; dotted line: control group) who reach the anatomical outcome (complete regression of any retinal fluid at SD-OCT), during the follow-up. At baseline (time 0), all patients have evidence of IRF/SRF. Starting from day 30, the solid line begins to deflect, as some patients in the treatment group start to show a condition of dry macula. The difference between the two groups is greatest at 60 days (*p*=0.01) and continues to be significant at 6 months (*p*=0.049).

Complete regression of IRF/SRF during the follow-up was evident in 8 eyes (72.7%) in the treatment group: 1 (12.5%), 6 (54.5%), and 1 (12.5%) eyes reached the outcome at 1, 2, and 3 months, respectively. Considering the eyes that presented with a dry macula at the second month, 2 (33.3%) still did not have any fluid at month three, while the other 4 (66.6%) showed a mild reactivation. All the eyes showed signs of recurrence at month 6. Only 2 (40%) eyes in the control group reached the outcome at month 3, with recurrence at month 6. Comparison between the groups showed that a significantly higher rate of the eyes in the treatment group reached the outcome (*p*=0.049) (Figure 1).

In both groups, median BCVA did not change significantly during the follow-up, as compared to baseline, and no differences between the two groups were found in final BCVA (Table 3). A median loss of 6 and 15 ETDRS letters in the treatment group (from 44 to 38, *p*=0.40), and in the control group (from 65 to 50, *p*=0.29), respectively, were reported at the end of the follow-up.

Median CFT and macular volume showed a reduction from the baseline in both the groups (Table 4). The macular volume significantly decreased only in the treatment group (Table 4).

FA at month 2 showed a reduction in the leakage area in both groups as compared to that in the baseline, reaching a statistically significant level only in the study group. Between-group differences in the extension of the leakage area were not significant (*p*=0.17) (Table 5).

Mean IOP increased throughout the follow-up period in the treatment group and returned to the baseline value (15 mmHg; range 12–16; *p*=0.52) at month 6 (Table 6). In three eyes (27.3%) from this group, IOP raised to 25 mmHg or more at least once during the study period (in 2 patients at day 15, in one patient at months 2 and 3). Two eyes that showed an IOP increase ≥10 mmHg were successfully treated with medical therapy (dorzolamide hydrochloride 20 mg + timolol maleate 5 mg ophthalmic solution). By contrast, in the control group, IOP was stable, never showing significant fluctuation until the end of the study (Table 6).

Cataract progression was evident in one eye (9%) in the study group, but surgery was not necessary. We did not report any other serious ophthalmic or systemic adverse event.

**Figure 2:**
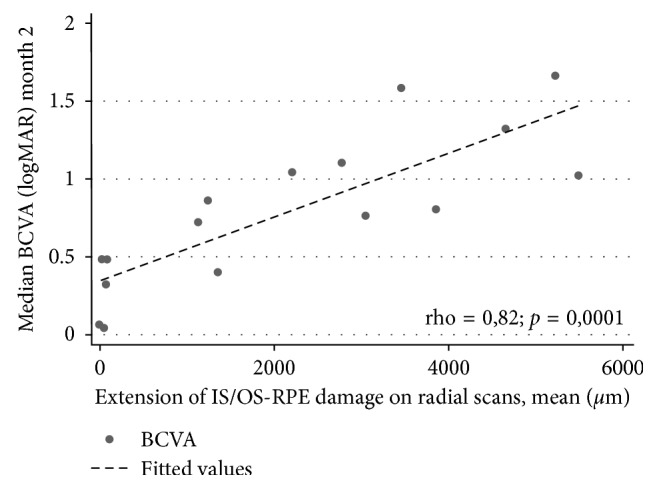
Scatter plot showing the correlation between the median BCVA at month 2 and the extension of the IS/OS junction, ELM, and RPE damage at 2 months (rho = 0, 82; *p*=0.0001).

BCVA at the second month significantly correlated with the extension of IS/OS junction, ELM, and RPE damage at month 2 (rho = 0.82; *p*=0.0001) (Figure 2), with the pattern of retinal fluid at month 2 (rho = −0.59, *p*=0.02) and with CNV type (rho = 0.63; *p*=0.008) at baseline. The extension of IS/OS junction, ELM, and RPE damage significantly correlated with the pattern of retinal fluid (rho = 0.73, *p*=0.001) and with the type of CNV lesion (rho = 0.6860, *p*=0.0033). No significant correlation between BCVA and median CFT at baseline (rho = 0.42; *p*=0.10), duration of the pathology (rho = 0.10; *p*=0.70), and pattern of nonresponse (rho = −0.06; *p*=0.8) was reported.

## 4. Discussion

In this pilot study, we evaluated the anatomical, functional efficacy and safety of a combined treatment with DXI and anti-VEGF in patients with wet-AMD and persistent retinal fluid despite conventional treatment. Partial results of this study were published elsewhere [13].

To date, there is no consensus on how many anti-VEGF injections should be administered before an AMD eye can be defined as a nonresponder. Some authors have defined as the nonresponders eyes that did not show a complete anatomical or functional improvement after at least 6 injections [14]. According to other reports, the eyes that fail to respond at the loading phase can be considered as early nonresponders [15]. In our study, we enrolled only the eyes that received at least 4 consecutive monthly injections of anti-VEGF agents before the screening examination, without showing a complete response.

We found that the combined therapy was beneficial since 72.7% of the eyes in the treatment group achieved a completely dry macula at least once during follow-up versus 40% of the eyes in the control group. Most of our cases, treated with a combined therapy, showed a complete resolution of CNV activity at the second month. This timing is consistent with former reports [16].

FA showed a significant reduction of the leakage area in the treatment group but not in the control group, thus providing evidence that OCT changes were a direct consequence of a reduction of CNV activity. By contrast, median BCVA did not improve significantly by the end of the follow-up in both the groups. The discrepancy between the dramatic anatomical improvement and the little change in BCVA may be explained by the existence of extensive damage to photoreceptors, ELM, and RPE (Figure 2).

The worst visual results were evident in the eyes with a preferential IRF pattern rather than those with SRF, and this correlation was also significant. A subanalysis of the CATT study showed less visual improvement in the eyes with evidence of intraretinal cysts [17]. We speculate that the lack of visual recovery in our patients with intraretinal cysts, despite resorption of fluid after the combined treatment, may be related to a preexisting irreversible outer retinal damage due to the long-lasting IRF [18].

Geographic atrophy (GA) is another known cause of reduced visual improvement in patients undergoing several intravitreal anti-VEGF injections [2]. FAF, performed at baseline and at the end of follow-up, suggested the existence of a certain degree of geographic atrophy in some of our patients. As already reported by other authors, the reliability and reproducibility of FAF for quantitative assessment of both the presence and the modification of the areas of GA are quite poor in patients with concomitant CNV [1]. For this reason, we did not evaluate the role of GA on pretreatment and final BCVA, nor its modification over time.

Inflammation has been shown to be a key element in the pathogenesis of AMD. Inflammatory cells, including macrophages and lymphocytes, have been found to arise in the affected area in the retina, together with neovascularization [5]. Cytokines and enzymes secreted by active inflammatory cells can damage Bruch's membrane, hence promoting neovascular membrane growth in the subretinal space [19]. Corticosteroids are known for their anti-inflammatory and antiangiogenic activity; they counteract macrophages and related cytokines involved in inflammation and neovascularization [20], modulate signaling pathway and effector proteins downstream of the VEGF receptor [21], restore the blood retinal barrier (BRB) through the induction of tight-junction proteins (occludin and claudin-5) [22], and modulate active microglia [23]. Additional corticosteroids seem to have the ability to target chronic inflammation when combined with anti-VEGF. Furthermore, the combination of intravitreal steroids with anti-VEGF agents may partially alleviate the efficacy decrease associated with repeated intravitreal anti-VEGF injections (tachyphylaxis) [5, 24].

Nevertheless, intravitreal steroids are associated with higher risk of ocular side effects, in comparison with intravitreal anti-VEGF agents [11, 12]. We did not observe any serious ocular or systemic side effect during the entire follow-up. A significant IOP increase was seen only in the treatment group. All cases of postoperative IOP increase were successfully managed with medical therapy alone. Our data on the safety profile of intravitreal DXI are in line with those reported by other authors [11, 12].

To the best of our knowledge, two other prospective studies on the use of DXI combined to anti-VEGF in recurrent or resistant cases of wet-AMD have been reported in the literature [5, 25]. In the first report, not only cases refractory to anti-VEGF therapy but also recurrent to wet-AMD cases were considered. This study demonstrated an overall reduction of ranibizumab retreatments in the combined therapy group, compared with the ranibizumab monotherapy group, with consistent functional outcomes [5]. Barikian et al. conducted a prospective uncontrolled study to assess the role of intravitreal DXI implant, combined with ranibizumab, in 19 neovascular AMD eyes resistant to at least three consecutive monthly injections of bevacizumab followed by at least three monthly ranibizumab. Complete retinal fluid resorption was reported in 13 eyes (68.4%) with intraretinal fluid, after 1 month.

Mean central retinal thickness significantly decreased at month 1, while mean BCVA did not change significantly. These authors concluded that intravitreal DXI implant effectively reduced intraretinal but not subretinal fluid in the eyes with neovascular AMD resistant to anti-VEGF [25]. By contrast, we did not observe any correlation between treatment efficacy and pattern of retinal fluid in our series. Other authors investigated the use of intravitreal DXI, combined with intravitreal anti-VEGF, in a mixed population of AMD patients, not specifically addressing nonresponders. Their findings suggested a possible advantage of the combined therapy over the monotherapy, in terms of a less need for retreatment, without significant additional effect on the visual function [26, 27].

The most important limitation of our study is the small sample size. However, as this was a pilot study, the patient number was small and sample size planning to ensure an adequate power was not necessary. The small sample size is at least in part due to the difficulty to find true nonresponders to anti-VEGFs among AMD patients. A follow-up of six months was too short to assess the true benefit and potential side effects of the combined treatment in the long run. However, this was beyond the original purpose of our study. Data from clinical study suggest that the activity of intravitreal DXI lasts approximately 3-4 months from implantation [11, 28]. We administered DXI only once at baseline, so we were not able to assess the effect of a second injection in patients who presented with a reactivation at month 6.

The points of strength of our study are, first of all, the prospective design and the presence of the control group in which the best anti-VEGF protocol (monthly injections) was performed. Furthermore, we only enrolled patients with a history of unresponsiveness to at least four consecutive monthly intravitreal injections of anti-VEGF, documented by SD-OCT scan, and we excluded patients with recurrent CNV. We assessed the anatomical success of our therapy, considering different OCT parameters, such as evidence of any retinal fluid, variation of CFT, and macular volume. We also analyzed the status of photoreceptors, ELM, and RPE and the extension of any damage in these layers, involving the fovea.

In conclusion, our data suggest that the dexamethasone intravitreal implant combined with anti-VEGF therapy may be a feasible option for those patients who show an incomplete response to anti-VEGF monotherapy, at least in terms of complete regression of retinal fluid. Larger studies are necessary to validate our findings and to verify if a prompter recourse to this treatment can translate into better visual outcome.

## Figures and Tables

**Table 1:** Exclusion and inclusion criteria.

Exclusion criteria
Retinopathy other than AMD
Uncontrolled glaucoma (IOP ≥ 25 mmHg)
NVG
Active inflammation and/or infection in the study eye
History of vitrectomy at any time
Cataract surgery within the previous 3 months
Ongoing therapy with other systemic or intravitreal steroids
Other previous treatment for wet-AMD

*Retreatment criteria*
BCVA loss ≥5 ETDRS letters
Recurrence or persistence of any fluid in the macula on SD-OCT
A 10% increase in CSFT in comparison with the previous value
New macular hemorrhages
New area of classic CNV
Development of new retinal PED or increase in size of an already existing PED

AMD: age-related macular degeneration; IOP: intraocular pressure; NVG: neovascular glaucoma; BCVA: best corrected visual acuity; ETDRS: early treatment diabetic retinopathy study; SD-OCT: spectral domain optical coherence tomography; CSFT: central subfield foveal thickness; CNV: choroidal neovascularization; PED: pigment epithelium detachment.

**Table 2:** Demographic characteristics of patients enrolled in the study.

Variables		Treatment	Control	*p* value
Age (SD)		73.2 (8.7)	79.2 (8.4)	0.22
Right eyes, *n* (%)		6 (54.6)	2 (40.0)	1
CNV type, *n* (%)	1	3 (27.3)	2 (40.0)	1
	2	4 (36.4)	1 (20.0)	
	Mixed	4 (36.4)	2 (40.0)	
Duration of AMD prior to randomization (months), median (range)		18 (12–48)	12 (6–18)	0.21
Number of previous IVT anti-VEGF, mean (SD)		8.8 (4.0)	9.2 (4.1)	0.86
Period of treatment with anti-VEGF before entering into the study (months), median (range)		13 (6–16)	11 (8–51)	0.69
OCT-CFT after last anti-VEGF injection before entering into the study (*μ*m), mean (SD)		387.6 (138.7)	488.2 (161.9)	0.22
OCT mean macular volume after the last anti-VEGF injection before entering into the study (mm^3^), median (range)		7.5 (7.3–7.7)	10.6 (8.8–11.1)	0.18
BCVA study eye (ETDRS letters), median (range)		44 (4–70)	65 (50–69)	0.33
BCVA study eye (logMar), median (range)		0.82 (0.30–1.62)	0.40 (0.32–0.70)	0.33
BCVA study eye (Snellen), median (range)		20/132 (20/40–20/833)	20/50 (20/42–20/100)	0.33
Pre-op IOP (mmHg), median (range)		15 (14–17)	14 (14–16)	0.6
OCT-MT (*μ*m), median (range)		462 (395–600)	354 (279–373)	0.10
OCT mean macular volume (mm^3^), median (range)		9.7 (8.3–9.8)	8.3 (8.0–8.4)	0.23
FA leakage area (mm^2^), median (range)		2.5 (0.5–5.8)	3.4 (1.1–6.2)	0.6

SD: standard deviation; range: minimum and maximum value; CNV: choroidal neovascularization; AMD: age-related macular degeneration; IVT: intravitreal therapy; VEGF: vascular endothelial growth factor; OCT: optical coherence tomography; CFT: central foveal thickness; BCVA: best corrected visual acuity; FA: fluorescein angiography.

**Table 3:** Median BCVA change during the follow-up and proportion of eyes with BCVA improvement ≥2 ETDRS lines, stable BCVA (change < 2 ETDRS lines), and BCVA decrease ≥2 ETDRS lines.

Median (range) BCVA value (logMAR)					
	Treatment group		Control group		Comparison between groups
Screening	0.82 (0.30–1.62)	—	0.4 (0.32–0.70)	—	*p*=0.33
15 days	0.92 (0.24–1.54)	*p*=0.23	0.46 (0.34–0.82)	*p*=1	*p*=0.46
1 month	0.82 (0.40–1.30)	*p*=0.75	0.40 (0.40–0.66)	*p*=1	*p*=0.31
2 months	0.86 (0.48–1.32)	*p*=0.55	0.48 (0.40–0.72)	*p*=0.25	*p*=0.17
3 months	0.90 (0.44–1.34)	*p*=0.33	0.54 (0.38–0.68)	*p*=0.35	*p*=0.11
6 months	0.92 (0.6–1.54)	*p*=0.40	0.7 (0.7–0.84)	*p*=0.29	*p*=0.42
BCVA change (6 months)	Eyes (%)		Eyes (%)		
BCVA improved (≥2 ETDRS lines)	9.1		0		
BCVA stable (change <2 ETDRS lines)	72.7		60		
BCVA worsened (≥2 ETDRS lines)	18.2		40		

**Table 4:** Median CFT and macular volume variation during the follow-up.

	Screening	15 days	1 month	2 months	3 months	6 months
*Median (range) CFT value (µm)*						
Treatment group	462 (395–600)	474 (368–636)	502 (366–651)	322 (227–509)	380 (191–566)	335 (250–609)
	—	*p*=0.96	*p*=1.00	*p*=0.006	*p*=0.08	*p*=0.27
Control group	354 (279–373)	296 (244–339)	330 (256–359)	334 (262–336)	318 (267–330)	292 (243–336)
	—	*p*=0.04	*p*=0.04	*p*=0.04	*p*=1.04	*p*=0.23
Comparison between groups	*p*=0.10	*p*=0.02	*p*=0.047	*p*=0.87	*p*=0.40	*p*=0.34

*Median macular volume value (mm* ^*3*^)						
Treatment group	9.7 (8.3–9.8)	9.04 (8.12–9.67)	9.0 (8.1–10.7)	8.09 (7.73–8.95)	8.78 (7.92–9.31)	8,45 (7,8–9,63)
	—	*p*=0.23	*p*=0.23	*p*=0.001	*p*=0.03	*p*=0.03
Control group	8.3 (8.0–8.4)	8.09 (7.91–8.26)	8.2 (7.9–8.4)	8.19 (8.11–8.20)	8.30 (8.09–8.40)	8,24 (8,09–8,3)
	—	*p*=0.06	*p*=0.38	*p*=0.38	*p*=0.50	*p*=0.22
Comparison between groups	*p*=0.23	*p*=0.16	*p*=0.23	*p*=0.95	*p*=0.46	*p*=0.28

IQR: interquartile range; CFT: central foveal thickness.

**Table 5:** Median area of leakage measured at the FA during the follow-up.

Median (range) area of leakage measured at the FA (mm^2^)		
	Screening	2 months
Treatment group	2.5 (0.5–5.8)	0 (0–0.67), *p*=0.01
Control group	3.4 (1.1–6.2)	0.73 (0.13–0.77), *p*=0.13
Comparison between groups	*p*=0.6	*p*=0.17

FA: fluorescein angiography.

**Table 6:** Median IOP variation during the follow-up.

Median (range) IOP variation (mmHg)						
	Screening	15 days	1 month	2 months	3 months	6 months
Treatment group	15 (14–17)	18 (16–22)	18 (17–19)	19 (16–20)	17 (14–19)	15 (12–16)
	—	*p*=0.11	*p*=0.04	*p*=0.11	*p*=0.19	*p*=0.52
Control group	14 (14–16)	15 (14–16)	14 (14–15)	17 (16–18)	16 (16–16)	15 (14–15)
	—	*p*=1	*p*=1	*p*=0.38	*p*=0.78	*p*=0.78
Comparison between groups	*p*=0.6	*p*=0.06	*p*=0.02	*p*=0.19	*p*=0.18	*p*=0.95

## Data Availability

The data used to support the findings of this study are available from the corresponding author upon request.
